# The Clinically Meaningful Link Between Alcohol Use and Attention Deficit Hyperactivity Disorder

**Published:** 2002

**Authors:** Bradley H. Smith, Brooke S.G. Molina, William E. Pelham

**Affiliations:** Bradley H. Smith, Ph.D., is an assistant professor in the Department of Psychology, University of South Carolina, Columbia, South Carolina. Brooke S.G. Molina, Ph.D., is an assistant professor in the Departments of Psychiatry and Psychology, University of Pittsburgh Medical Center, Pittsburgh, Pennsylvania. William E. Pelham Jr., Ph.D., is a professor in the Departments of Psychology, Pediatrics, and Psychiatry, State University of New York at Buffalo, Buffalo, New York

**Keywords:** attention deficit disorder with hyperactivity, alcohol and other drug dependence, alcoholic beverage, comorbidity, impulsive behavior, symptom, early AODU (alcohol and other drug use) onset, conduct disorder, diagnostic criteria, causal pathways, predictive factor, treatment outcome, correlation analysis, literature review

## Abstract

Attention deficit hyperactivity disorder (ADHD) is a childhood mental health disorder that can lead to alcohol and other drug (AOD)-related problems if it persists into adolescence and adulthood. Several findings suggest that ADHD contributes to the development of AOD use disorders. ADHD generally precedes alcohol use and is correlated with developmentally inappropriate levels of alcohol use or abuse; conduct problems typically precede the development of alcohol use or abuse. The potential role of ADHD in the development of AOD use problems has important implications for prevention and treatment of such problems. For example, people with ADHD have poor outcomes from AOD abuse treatment. Service providers who work in AOD abuse treatment settings must develop the diagnostic and clinical expertise to address co-occurring ADHD and AOD use disorders.

Attention deficit hyperactivity disorder (ADHD) is a childhood mental health disorder that is characterized by inattention, impulsivity, and hyperactivity. It has been discussed in the psychiatric literature for approximately 100 years under various names, including minimal brain dysfunction; hyperkinetic reaction of childhood; attention deficit disorder; and, since 1987, ADHD. The various names reflect an evolving understanding of the disorder and an increasing consensus on its nature. One of the major recent advances in the research on ADHD is the recognition that most people who are diagnosed with it as children continue to suffer problems related to this disorder as adolescents and adults (Barkley 1998; Tucker 1999). This recognition that ADHD extends beyond puberty has expanded ADHD research into new areas, including the link between ADHD and alcohol use and abuse.

To provide the appropriate background for understanding the link between ADHD and alcohol use, this article first summarizes the diagnostic criteria for ADHD. It then examines the hypothesis that ADHD is a causal factor in the development of problematic alcohol consumption. Finally, the article reviews some preliminary research suggesting that ADHD and its treatment may have important implications for the prevention and treatment of alcohol-related problems.

## Diagnosis of ADHD

According to the American Psychiatric Association (APA) (2000), the rate of ADHD in the general population of children in the United States is between 3 and 7 percent. Among children receiving treatment for psychiatric disorders in clinical settings, however, this rate often exceeds 50 percent (Barkley 1998), making ADHD one of the most commonly diagnosed psychiatric disorders for children in the United States. The rate of ADHD in adults is thought to be somewhat lower than in children, probably between 2 and 5 percent in the general population (Barkley 1998). For adults in clinical settings, the rate of ADHD is currently unknown but appears to be substantially elevated compared with the general population. Among adult patients receiving treatment for alcohol and other drug (AOD) abuse, the rate of ADHD has been estimated to be approximately 25 percent (Wilens 1998). A similar rate of ADHD, about 30 percent, has been found among adolescents in treatment for AOD use disorders (AODD) (Molina et al. 2002). These relatively high rates indicate that it is important for service providers in AOD-abuse treatment settings to be able to diagnose and treat ADHD.

The symptoms or problems associated with ADHD vary somewhat depending on the patient’s developmental stage (Barkley 1998). Children with ADHD typically present with academic difficulties, discipline problems at school and at home, and conflicts with peers. Adolescents with ADHD exhibit many of the same problems but often with more serious consequences, such as dropping out of school or experiencing legal problems. Moreover, because of their physical and social maturation, adolescents with ADHD encounter new sets of problems, such as sexual activity and pregnancy and AODD. In adults with ADHD, school-related problems may no longer be relevant, but social problems often persist and new challenges may develop related to driving (e.g., traffic violations), vocational achievement, and sustaining friendships and romantic relationships.

The most recent set of criteria for a diagnosis of ADHD as listed in the *Diagnostic and Statistical Manual of Mental Disorders, Fourth Edition, Text Revision* (APA 2000) (see textbox) reflects the theory that ADHD is made up of two major dimensions: inattention and hyperactivity/impulsivity. Overall, the symptoms associated with ADHD fall into three categories—inattention symptoms, hyperactivity symptoms, and impulsivity symptoms. Factor analytic studies^1^ have found that inattention symptoms represent a single dimension that is reasonably distinct from hyper-activity and impulsivity (e.g., Lahey et al. 1988; Molina et al. 2001). Hyper-activity symptoms and impulsivity symptoms, however, are not distinct from each other and combine to form a dimension that is separate from inattention (Barkley 1998; Milich et al. 2002). Because symptoms from both dimensions can co-occur, however, three major subtypes of ADHD exist:

- ADHD, predominantly inattentive subtype (ADHD–IA)
- ADHD, predominantly hyperactive/impulsive subtype (ADHD–HI)
- ADHD, combined inattentive and hyperactive/impulsive subtype (ADHD–C).

**Diagnostic Criteria for Attention Deficit Hyperactivity Disorder (ADHD)**

**To be diagnosed with ADHD, a person must:**

- Meet the criteria for one of the three subtypes of ADHD:
  1. Predominantly Inattentive type: six symptoms of inattention, as listed below
  2. Predominantly Hyperactive/Impulsive type: six symptoms of hyperactivity/impulsivity, as listed below
  3. Combined type: six symptoms from each of the lists below
- Have symptoms that have persisted for at least 6 months and which must be present to a degree that creates problems and is inconsistent with developmental level
- Have at least some symptoms that caused impairment before the age of 7
- Have some symptoms that are present in at least two different settings (e.g., home, school), and that cause clinically significant impairment in social or academic functioning
- Have symptoms that are not associated with another disorder (e.g., psychotic disorder, mood disorder, personality disorder, or anxiety disorder).

**A person with Predominantly Inattentive ADHD often:**

- Fails to give close attention to details or makes careless mistakes
- Has difficulty sustaining attention in tasks or play activities
- Does not seem to listen when spoken to directly
- Does not follow through on instructions and fails to finish tasks (not as a result of oppositional behavior or failure to understand instructions)
- Has difficulties organizing tasks and activities
- Avoids or dislikes tasks that require sustained mental effort
- Loses things necessary for tasks or activities (e.g., toys, books)
- Is easily distracted
- Is forgetful in daily activities.

**A person with Predominantly Hyperactive/Impulsive ADHD often:**

- Fidgets with hands or feet or squirms in seat
- Leaves seat in classroom or in other situations in which remaining seated is expected
- Runs or climbs excessively when it is inappropriate
- Has difficulty playing or engaging in leisure activities quietly
- Is always “on the go” or acts as if “driven by a motor”
- Talks excessively
- Blurts out answers before questions have been completed
- Has difficulty awaiting turn
- Interrupts or intrudes on others (e.g., interrupts conversations or games).

SOURCE: Adapted from the *Diagnostic and Statistical Manual of Mental Disorders, Fourth Edition, Text Revision* (American Psychiatric Association 2000).

Children with ADHD–IA show a different impairment profile than do children with either ADHD–HI or ADHD–C (Barkley 1998; Milich et al. 2002). Thus, children with ADHD–IA typically exhibit sluggish information processing, academic problems, and social neglect (e.g., they ignore or are ignored by their peers). Conversely, children with ADHD–HI or ADHD–C exhibit deficits in behavioral response inhibition that often result in careless mistakes, impulsive rule breaking, and conflict with peers and adults. Distinctions between the different subtypes of ADHD may have important implications for the effective treatment of the disorder.

The characteristics of patients with ADHD differ not only among patients with the ADHD subtypes specified above but also between adults on the one hand and children and adolescents on the other. For instance, the ratio of males to females in treatment for ADHD in childhood is approximately 8 to 1. Based on preliminary research, however, this ratio appears to be 1:1 in the adult population (Biederman et al. 1993). The reasons for the change in gender ratio are poorly understood, and this phenomenon warrants replication and further study. Nevertheless, the change in gender ratio suggests that there are substantial differences in referral patterns between children and adults, with males receiving much more treatment in childhood compared with females. These differences in referral patterns have several implications for the interpretation of scientific studies. For example, longitudinal studies of ADHD that began when subjects were children provide less information about the link between ADHD and alcohol use for females than for males.

From a clinical standpoint, when diagnosing ADHD in adulthood it is important to establish that the impairment required for a diagnosis is the result of core symptoms of ADHD rather than of coexisting disorders, such as depression or alcoholism. Also, adults are more prone to metabolic conditions, such as hyper-thyroidism,^2^ that might result in symptoms or impairments similar to those associated with ADHD. Accordingly, a careful physical exam is warranted for adult patients before establishing a diagnosis of ADHD (Wilens 1998). Because it can be difficult to disentangle the origin of impairment, determining the age of onset of ADHD symptoms and the related impairment is crucial for a correct diagnosis.

For a diagnosis of ADHD, the problems must be traced back to developmentally inappropriate levels of inattention or hyperactivity/impulsivity, and the person must exhibit the minimum number of symptoms on at least one dimension of ADHD (see textbox). Furthermore, these symptoms must cause impairment in two or more settings and contribute to problems before age 7, and must not be attributable to another psychiatric or medical disorder (APA 2000). As with all mental health disorders, no single medical or psychological test can confirm or refute a diagnosis. Instead, information is gathered from multiple sources and combined to determine if the diagnosis is appropriate.

A proper evaluation is time consuming, typically involving several hours of assessment by a mental health professional. Appropriate screening tests, such as those published in Barkley and Murphy’s (1998) *Clinical Workbook*, may reduce this time demand. However, such tests are useful only when the number of positive responses required to indicate a diagnosis is reasonably low in order to avoid “false negatives”—cases in which a client has a disorder but does not show up as positive for the disorder on the test. The ability of screening tests for ADHD to avoid false negatives is particularly important because adolescents and adults typically underreport symptoms of ADHD (Smith et al. 2000; Barkley et al. 2002). Accordingly, clinicians cannot confidently rule out ADHD until they receive collateral information (e.g., in the form of ratings) from parents; teachers; or other reliable sources, such as roommates or significant others.

State-of-the-art assessments for ADHD in children typically include standardized rating scales from parents and teachers; interviews with parents; and collateral information, such as school records. In adults, the diagnosis is typically based on an interview with the adult, which can be supplemented with collateral interviews with parents or a romantic partner or roommate. As mentioned above, such collateral information for adult clients is highly desirable, especially when the clinician must retrospectively reconstruct a timeline of symptoms and dysfunction indicative of ADHD. Diagnosis in adults also would be aided by psychometrically sound, developmentally appropriate rating scales, similar to those made for children, that have self- and collateral-report formats. Most scales for adults, however, are still being developed or modified, and clinicians should use them with caution until research supports their validity, especially for the self-report rating scales. Once valid scales become available, they will help the clinician establish whether symptoms are at a developmentally inappropriate extreme.

Another factor complicating the diagnosis of ADHD in adults is that the current diagnostic criteria are based on a symptom list that was developed for children and may not be entirely appropriate for adults. For example, symptoms of hyperactivity tend to decrease with age; therefore, adults rarely exhibit such symptoms as “running or climbing at inappropriate times.” Consequently, fewer symptoms pertain to adults, making it more difficult to achieve a diagnosis during adulthood. Barkley (1998) therefore has suggested that for adults, the threshold of symptoms required for a diagnosis be lowered to four or five of nine symptoms of either inattention or hyperactivity/impulsivity, rather than the six symptoms that are required for a diagnosis of ADHD in children. This modification has not been formally accepted, and as a result, many adults are diagnosed with ADHD in partial remission (APA 2000).

## The Causal Link Between ADHD and Alcohol Use and Abuse

Three conditions must be met to demonstrate that ADHD causes alcohol-related problems. It should be noted that none of the individual conditions *proves* causation. All three conditions must be met simultaneously to provide the minimal conditions for demonstrating causation. Also, these are only the three *minimum* conditions for demonstrating causation. As discussed below, other conditions or considerations may corroborate or contradict a causal hypothesis. The three minimum conditions for demonstrating a causal link between ADHD and alcohol are:

- The cause (i.e., ADHD) must precede the effect (i.e., alcohol consumption).
- ADHD and alcohol use, abuse, or related problems must be correlated, and the correlation should be large enough to mandate consideration from a theoretical or applied standpoint.
- ADHD must be a unique cause of alcohol-related problems, independent of other variables that might plausibly cause those problems.

A fourth consideration that is helpful, but not required, is to have a theory of the mechanisms that mediate the relationship between ADHD and alcohol use. The following sections discuss data that pertain to the three necessary conditions for a causal effect. Potential mechanisms that might mediate or influence such a causal relationship are discussed in the section “Mediators Between ADHD and Alcohol Use and Abuse.”

### Timeline of ADHD and Alcohol Use

Given that a formal diagnosis of ADHD requires that problems are evident before age 7 and that 7-year-olds almost never exhibit any deviant behavior related to alcohol, the requirement that cause precede effect (i.e., that ADHD precede alcohol use) is easily satisfied. It is noteworthy that the typical length of time between recognition of ADHD and the onset of drinking provides ample opportunity for interventions to prevent alcohol-related problems. Some non-pharmacological interventions for children and adolescents with ADHD and their families, such as training in effective parenting, hold promise for reducing rates of alcohol-related problems among people with ADHD (e.g., Robbins and Szapocznik 2000). The role of pharmacological treatment of ADHD in preventing future substance abuse is a matter of debate. Despite some promising initial results (e.g., Biederman et al. 1999), the long-term effects of pharmacological treatment of ADHD are poorly understood (Pelham et al. 1998).

### Correlation Between ADHD and Alcohol Use

The second condition for inferring that ADHD contributes to alcohol use and abuse requires that ADHD and alcohol use be correlated. It is important to note, however, that correlation does not prove causation, but causation implies correlation. Therefore, correlation is one of the three minimum conditions for inferring a causal link between ADHD and alcohol use.

At first glance, the evidence for a correlation between ADHD and alcohol use is mixed. However, careful examination of data regarding alcohol use in early adolescence and alcohol abuse and dependence in early adulthood suggests that there is a meaningful correlation between ADHD and alcohol use.

In one study in which children diagnosed with ADHD and control children were followed for 8 years (i.e., a prospective longitudinal study), investigators found that at a mean age of 14.9 years, 40 percent of the ADHD children, but only 22 percent of the control children, had used alcohol (Barkley et al. 1990). This finding suggests that ADHD is associated with an early onset of alcohol use. In contrast, a study in young adults (mean age of 25 years) found no difference in alcohol use between people with ADHD (92 percent) and without ADHD (95 percent) (Weiss and Hechtman 1993). These rates are similar to the rate of alcohol use in the general population and have little clinical meaning. Thus, taken together without consideration of the age of the participants, these studies as well as others suggest that the evidence for a correlation between ADHD and alcohol use is mixed and possibly weak. However, one can argue that alcohol use is a “normal” behavior for young adults and has only limited predictive power for alcohol-related problems later in life. Conversely, early onset of alcohol use during adolescence is a strong predictor of AOD-related problems later in life (Clayton 1992). Accordingly, the implications of alcohol use in early adolescence may be more important than the implications of alcohol use during young adulthood.

Although elevated rates of alcohol *use* during adolescence and young adulthood do not necessarily imply that people with ADHD experience more problems, elevated rates of alcohol use *disorders* (i.e., alcohol abuse and dependence) are clear signs of a link between ADHD and AOD-related problems. Therefore, researchers have investigated the rates of alcohol use disorders in people with and without ADHD. One study of adolescents with ADHD (mean age 14.4 years) found no statistically significant differences in the rate of alcohol use disorder diagnoses (i.e., 15 percent) compared with a matched control group (i.e., 16 percent) (Biederman et al. 1997). In a study of young adults (mean age 25), however, approximately 44 percent of the participants with ADHD met the criteria for alcohol abuse or dependence, compared with 27 percent of the control participants (Weiss and Hechtman 1993). Because the same study found that the rates of alcohol use were similar for young adults with and without ADHD, these findings indicate that those with ADHD may be more likely to use alcohol excessively compared with people without the disorder. It is also noteworthy that the transition from AOD use to abuse is more rapid for people with ADHD than for people without the disorder and that AODDs appear at earlier ages in people with ADHD (i.e., at age 19) than in people without ADHD (i.e., at age 22) (Wilens 1998). Although these findings support the link between ADHD and alcohol-related problems, other longitudinal studies have contradicted these findings (e.g., Lambert and Hartsough 1998; Mannuzza et al. 1993) and this issue warrants further study.

The fact that alcohol use disorder rates do not differ between adolescents with and without ADHD is possibly an indication that the diagnostic criteria for alcohol use disorders may not be developmentally appropriate for adolescents (Bukstein and Kaminer 1994). It might be more appropriate to use measures of heavy drinking rather than AOD diagnoses in studies of adolescents. For example, the concept of “binge drinking” (i.e., consumption of five or more drinks on one occasion) might identify people at elevated risk for alcohol-related problems or later AODD who in most cases do not currently meet diagnostic criteria for an AODD. Also, using a continuous measure (e.g., the typical number of drinks consumed per occasion) rather than a categorical measure (e.g., binge drinking or AOD diagnosis) might be more sensitive to differences in drinking patterns in adolescence (Bukstein and Kaminer 1994).

To summarize, it appears that a correlation exists between ADHD and AOD-related diagnoses but that this phenomenon is mostly evident during adulthood. Also, there appears to be a correlation between ADHD and alcohol use, but this is mostly evident during early adolescence. Further study using measures that are more age-appropriate or more sensitive to differences in populations, such as measures of heavy drinking, may be necessary to document a clear link between ADHD and alcohol use during mid- to late adolescence.

### ADHD as an Independent Predictor of Alcohol-Related Problems

The third condition required for ADHD to be considered a cause of alcohol-related problems is that the presence of ADHD independently (i.e., in the absence of other comorbid disorders) predicts those problems. The comorbid disorder that is most often implicated as a confound to the link between ADHD and alcohol is conduct disorder (CD), or more broadly, the antisocial spectrum of behavior that includes aggressivity and oppositional defiant disorder in childhood, CD in adolescence, and antisocial personality disorder (ASPD) in adulthood. In clinical samples of children with ADHD followed into adolescence, elevated rates of AODD were found only among those who had developed CD (e.g., Barkley et al. 1990; Gittleman et al. 1985). This finding among middle- and late-aged adolescents has led to conclusions that CD and not ADHD underlies AOD risk. However, there are other plausible explanations that do not preclude ADHD as a cause of AODD. For example, diagnostic criteria for CD and AODD may be confounded or the disorders may co-occur at such high rates during adolescence that they cannot be disentangled. Thus, ADHD could cause both CD and AODDs and therefore could still be a legitimate causal factor for AODDs. At present, such assertions are speculative, and appropriate analysis of longitudinal data will be necessary to untangle the complex causal relationships between ADHD, CD, and AODD.

In a comprehensive review of the literature on comorbidity between ADHD and AODD in adults, Tucker (1999) noted that the diagnosis of ADHD by itself appears to increase the risk for AODDs. However, Tucker also concluded that the elevation in risk was about the same magnitude as that associated with other psychiatric disorders, such as depression and anxiety. Even comorbidity of ADHD with other mental disorders was not associated with substantially elevated rates of alcohol-related problems in adults (Wilens 1998). The exceptions to this general finding are conduct disorder and antisocial personality disorder, because the risk for developing an AODD is substantially elevated when ADHD occurs in conjunction with one of these conditions (Tucker 1999). Co-occurring bipolar disorder also can possibly increase the risk of AODD (for more information on the relationship between bipolar disorder and AODD, see the article in this issue by Sonne and Brady, pp. 103–108).

Given the strong link between ADHD, CD, and AODD, it may be worthwhile to comment further on the relationship between CD and ADHD. CD is characterized by gross violations of social norms, such as irresponsibility, lying, criminality, and aggression. (For more information on CD and its association with alcohol use and abuse, see the article in this issue by Clark and colleagues, pp. 109–115.) Approximately 25 percent of children and 50 percent of adolescents with ADHD also meet the diagnostic criteria for CD (Barkley 1998). Furthermore, at least 70 percent of people with CD meet the diagnostic criteria for ADHD. Although most cases of CD are limited to the adolescent years, some people show a lifespan-persistent course that begins with a pattern of behavioral undercontrol in childhood consistent with the symptoms of ADHD (Moffitt 1990; Moffitt et al. 2002). People with early onset and persistent symptoms of CD often meet diagnostic criteria for ASPD, which is characterized by a callous disregard for the rights and needs of others. People with ASPD have the worst prognosis— that is, the greatest likelihood and greatest severity of AOD-related problems (Frick 1998; Moffitt et al. 2002). Therefore, determining the comorbidity of CD or ASPD with ADHD as well as the age of onset of CD symptoms may be important in preventing and treating alcohol-related problems.

It appears, then, that the link between ADHD and AODD is most pronounced in people with severe behavior problems. As a cautionary note, however, most of the research on this topic is based on studies with potentially significant selection biases. For example, any association between a psychiatric condition and AODD in clinical populations should be viewed cautiously because referral patterns may result in a selection bias for the more severe cases (Tucker 1999). Therefore it is likely that people with externalizing behavior problems (e.g., CD spectrum disorders) are over-represented in studies of adolescents, and people with internalizing disorders (e.g., anxiety and depression) could be over-represented in studies of adults. Studies that include more representative samples may find links between ADHD and AOD that are not necessarily associated with antisocial behavior. For instance, there could be at least two pathways from ADHD to AODD—that is, early onset AODD could be associated with hyperactivity/impulsivity (and possibly antisocial behavior) and later onset AODD could be associated with inattention (and possibly anxiety or depression).

## Mediators Between ADHD and Alcohol Use and Abuse

Assuming that a causal link exists between ADHD and alcohol use, an important issue related to selecting the most appropriate treatment for patients with ADHD and an AODD is that various factors may underlie, or mediate, the relationship between ADHD and AODD. Several such mediating factors have been suggested, including certain brain chemicals (described below), relative susceptibility to developing alcohol-related problems, and levels of sociopathy.

Clinical neuroscience has begun to identify some possible brain-based links between ADHD and alcohol use and abuse. Of particular interest is the dopamine hypothesis of ADHD (Solanto 2002) and the role of the medial fore-brain dopamine system in the development of AODDs (Hyman and Malenka 2001). Very briefly, the dopamine hypothesis of ADHD posits that low levels of the brain chemical dopamine in the forebrain cause problems with executive functions related to attention and impulse control. Support for this hypothesis comes from brain scan research and from the fact that many drugs that successfully treat ADHD raise levels of dopamine in the brain. This and other related hypotheses clearly support the notion that ADHD is a brain-based disorder rather than a phenomenon that is based solely on social expectations of what behavior is considered “normal.” In addition, these hypotheses may have important implications for the pharmacological treatment of ADHD (although it is important to stress that just because a disorder is brain-based does not mean that only pharmacological interventions are appropriate).

The dopamine system also has been implicated in mediating some of alcohol’s effects on the brain and therefore may play a role in the development of alcohol use disorders. Accordingly, disturbances in the dopamine system may underlie both ADHD and alcohol use disorders and may therefore contribute to the association between the two disorders.

Another possible mediating mechanism between ADHD and AODDs is that people with ADHD may have lower thresholds for AOD-related problems. For example, people with ADHD typically have low baseline levels of impulse control. As a result, they would experience alcohol’s detrimental effects (e.g., impaired driving ability) after consuming less alcohol (i.e., at lower blood alcohol levels) compared with people without ADHD. Similarly, greater levels of inattention associated with ADHD could augment alcohol’s detrimental cognitive effects.

It also is possible that people with ADHD use AODs in an effort to self-medicate distress related to ADHD or co-occurring conditions (Wilens 1998). Thus, it may be worthwhile for physicians to assess their clients’ levels of subjective distress and their beliefs about the costs and benefits of using AODs to relieve that distress.

Finally, children with ADHD commonly experience social deficits and academic problems, and these problems may play important roles in the mediational chain linking ADHD and alcohol problems. Appropriate intervention with parents and teachers to provide better social structure and academic support might prevent many problems related to ADHD, including the potential for developing an AODD (Clayton 1992).

Some researchers have argued that in people with co-occurring CD and ASPD there may be two mechanisms linking ADHD and alcohol use (Frick 1998). Both ADHD and sociopathy have been associated with high rates of paternal alcoholism and a family history of ASPD (Barkley 1998). In this case, indulgence of desires resulting from sociopathic irresponsibility may lead to higher rates of alcohol consumption and alcohol-related problems.

Alternatively, low behavioral control related to extreme impulsivity in people with ADHD and comorbid CD may account for elevated AODD problems. Thus, people with ADHD and comorbid CD might be regarded as “super impulsive.” To better understand these competing, but not mutually exclusive, mediating theories, research that measures and models sociopathy and impulsivity as separate dimensions is needed.

Understanding the unique treatment implications related to inattention, impulsivity, and callous/unemotional states may be one of the keys to unlocking the link between ADHD and alcohol use. Unfortunately, the current diagnostic criteria for CD muddle the distinction between impulsivity and callous/unemotional traits.

## ADHD and Alcohol-Related Interventions

Most of the available data suggest that ADHD has detrimental effects on the prevention and treatment of alcohol-related problems. For example, one of the most commonly used approaches in the prevention of AODD is cognitive therapy designed to improve self-control and appropriate problem solving. Unfortunately, these cognitive techniques do not work with children with ADHD (Pelham et al. 1998), their efficacy with adolescents with ADHD has barely been studied (Smith et al. 2000), and there is no data on this treatment in adults with ADHD. Hence, most programs to prevent AODDs have components that are ineffective with children and of unknown efficacy with adolescents or adults with ADHD. Consequently, in preventive interventions based on cognitive therapy, people with ADHD may require supplemental intervention that is specifically adapted to their unique needs to effectively prevent AODDs. Several promising psychosocial interventions have empirical support in children and adolescents with ADHD (Pelham et al. 1998; Smith et al. 2000). These include appropriate supervision; consistent enforcement of developmentally appropriate rules using behavioral contingencies;^3^ promoting success at school and with peers; and modeling of appropriate communication and behavior, especially regarding AOD use, by parents (Clayton 1992).

If prevention fails, people with ADHD who develop an AODD fare poorly in AODD treatment compared with peers who do not have ADHD. In one study, people with ADHD took more than twice as long to recover from AODDs than did people without ADHD (Wilens 1998). Similarly, Carroll and Rounsaville (1993) reported a poorer AODD treatment outcome for patients with untreated ADHD. One possible explanation for this phenomenon is that people with ADHD tend to exhibit a greater range of AOD-related problems (i.e., more severe AOD use) than do people without ADHD (e.g., Molina et al. 2002; Thompson et al. 1996), which, in turn, is associated with a poorer treatment outcome (Clayton 1992).

Another possible explanation for poor outcomes of patients with ADHD in AODD treatment is that, because of their core deficits of inattention and impulsivity, these patients may respond poorly to standard treatments based on cognitive therapy or semistructured group therapy. Effective treatment of the core symptoms of ADHD with stimulant medication may make patients more amenable to standard AODD treatment; however, stimulants work for approximately 70 percent of people with ADHD, leaving a substantial number of clients who may not respond to AODD treatment (Barkley 1998). Moreover, there is limited information and lack of consensus regarding the effects, either beneficial or detrimental, of stimulant treatment for adolescents or adults with ADHD and comorbid AODD. Some authors caution against stimulant treatment and suggest alternative pharmacological approaches (Riggs 1998). Others advocate the well-monitored use of stimulants (Wilens 1998).

In addition to treating the core symptoms of ADHD, another consideration is the high stress level observed in families of individuals with ADHD and the contribution of poor family functioning to poor AODD outcomes (Clayton 1992). There can also be indirect effects of ADHD on AODD treatment outcomes, such as when the stress of raising a child with ADHD might impair a parent’s recovery from AODD (Pelham and Lang 1993). Thus, there is a strong need for psychosocial interventions that simultaneously address issues related to ADHD and AODD. Some promising options that have not been tested in patients with both ADHD and AODDs but which have proven effective in the treatment of AODD are stimulating, flexible, and engaging interventions, such as brief, strategic family therapy (Robbins and Szapocznik 2000) and motivational interviewing (Miller 1998). Studies of these and other treatments may lead to a better understanding of the link between ADHD and alcohol use and, eventually, to better prevention and treatment of co-occurring ADHD and AODDs.

In summary, professionals working with clients with ADHD must be prepared for intra- and interpersonal dysfunction resulting from one or more common problematic symptom clusters, including high levels of inattention, impulsivity, or—among those with comorbid CD or ASPD—callous disregard for others. In addition, the specific problems that must be addressed as well as the appropriate therapeutic approaches most likely will differ among clients. Thus, people with the ADHD–IA subtype may have different history, prognosis, and treatment needs than do people with the ADHD–HI subtype. If comorbid CD or ASPD adds callous/unemotional traits to the clinical picture, these features also must be considered in the treatment plan. Research has not yet determined whether patients with different ADHD subtypes respond differently to treatment. It is clear, however, that ADHD patients with comorbid CD or ASPD have poorer outcomes (Barkley 1998).

## Summary and Conclusions

Clinical researchers and AODD service providers need to be prepared for co-occurring ADHD and AODD. The rate of ADHD is at least 25 percent among patients receiving treatment for AODDs, and 20 to 50 percent of adults diagnosed with ADHD meet criteria for an AODD. Furthermore, the rate of ADHD may be as high as 50 percent in high-risk populations^4^ targeted for prevention. The extent to which ADHD causes AOD problems is currently a matter of debate. However, ADHD clearly precedes AOD use, and therefore children and adolescents with ADHD may be a good target population for AOD prevention, especially if modifications are made to prevention programs to address the unique needs of individuals with ADHD. AODD treatment programs that offer standard clinical care for dually diagnosed adolescents and young adults also should provide appropriate evaluation and treatment of ADHD and related comorbid disorders, especially CD and ASPD. Treatment with medications such as stimulants needs more study in AODD populations but could help to overcome barriers to positive AOD treatment outcome that arise from core deficits of hyperactivity, impulsivity, and inattention. Psychosocial interventions should be tailored to the specific cognitive and interpersonal styles of clients with ADHD and should consider the likelihood that many of these patients are also using AODs. Promising, but as yet untested, interventions for such clients include brief strategic therapy and motivational interviewing.

## Footnotes

1 This type of study examines how sets of items form statistically distinct groups or dimensions that measure different characteristics.

2 Hyperthyroidism is a condition characterized by excessive activity of the thyroid gland that can lead to nervousness, tremors, constant hunger, weight loss, fatigue, or diarrhea.

3 Contingencies are consequences (i.e., rewards or punishment) for desired and undesired behaviors that are spelled out when setting up rules of behavior.

4 The term “high-risk” refers to people who exhibit risk factors predictive of developing a problem but who have not yet developed the problem (e.g., alcohol use). For example, children with learning and behavior problems are at elevated risk for AODD compared with most of their peers. Children with ADHD would be overrepresented among such a group. High-risk groups might need interventions that are more intensive or specially tailored than those given to the whole population.
